# DHA Inhibits Protein Degradation More Efficiently than EPA by Regulating the PPAR**γ**/NF**κ**B Pathway in C2C12 Myotubes

**DOI:** 10.1155/2013/318981

**Published:** 2013-07-28

**Authors:** Yue Wang, Qiao-wei Lin, Pei-pei Zheng, Jian-song Zhang, Fei-ruo Huang

**Affiliations:** Department of Animal Nutrition and Feed Science, College of Animal Science and Technology, Huazhong Agricultural University, Wuhan 430070, China

## Abstract

This study was conducted to evaluate the mechanism by which n-3 PUFA regulated the protein degradation in C2C12 myotubes. Compared with the BSA control, EPA at concentrations from 400 to 600 *µ*M decreased total protein degradation (*P* < 0.01). However, the total protein degradation was decreased when the concentrations of DHA ranged from 300 *µ*M to 700 *µ*M (*P* < 0.01). DHA (400 *µ*M, 24 h) more efficiently decreased the I**κ**B**α** phosphorylation and increased in the I**κ**B**α** protein level than 400 *µ*M EPA (*P* < 0.01). Compared with BSA, 400 *µ*M EPA and DHA resulted in a 47% or 68% induction of the NF**κ**B DNA binding activity, respectively (*P* < 0.01). Meanwhile, 400 *µ*M EPA and DHA resulted in a 1.3-fold and 2.0-fold induction of the PPAR**γ** expression, respectively (*P* < 0.01). In C2C12 myotubes for PPAR**γ** knockdown, neither 400 *µ*M EPA nor DHA affected the levels of p-I**κ**B**α**, total I**κ**B**α** or NF**κ**B DNA binding activity compared with BSA (*P* > 0.05). Interestingly, EPA and DHA both still decreased the total protein degradation, although PPAR**γ** knockdown attenuated the suppressive effects of EPA and DHA on the total protein degradation (*P* < 0.01). These results revealed that DHA inhibits protein degradation more efficiently than EPA by regulating the PPAR**γ**/NF-**κ**B pathway in C2C12 myotubes.

## 1. Introduction

Nuclear factor-kappa B (NF*κ*B) is one of the most important signaling pathways linked to the loss of skeletal muscle mass in normal physiological and pathophysiological conditions [1]. NF*κ*B expresses constitutively and exists in the cytosol as part of a heterotrimeric complex [2]. This complex typically comprises the DNA-binding proteins p50 and p65 plus the inhibitory protein I*κ*B*α*. Activation of NF*κ*B requires phosphorylation of I*κ*B*α*, followed by ubiquitin conjugation and proteolysis of I*κ*B*α* by the 26S proteasome [3, 4]. The activated NF*κ*B dimer is then translocated to the cell nucleus, which is a feed-forward signal that leads to the upregulation of pathway components including ubiquitin, E_2_/E_3_ proteins, and proteasome subunits, subsequently increasing activity of the ubiquitin/proteasome pathway and enhancing skeletal muscle protein degradation [5–7].

There is growing evidence suggesting that long chain eicosapentaenoic acid (EPA, C20:5n-3) can inhibit NF*κ*B activation by preventing the degradation of I*κ*B*α* in skeletal muscle [8, 9]. Remarkably, most of the previous researches focused on that EPA can decrease muscle protein degradation in cancer cachexia [10–12]. In addition, it was further observed that long chain EPA, but not *α*-linolenic acid (ALA, C18:3n-3), can inhibit the NF*κ*B activation in C2C12 myotubes by activating transcription factors peroxisome proliferators-activated receptor-*γ* (PPAR*γ*) [13]. The above results demonstrated that the effect of n-3 polyunsaturated fatty acid (n-3 PUFA) on the I*κ*B*α*/NF*κ*B pathway may be different because of the variety of fatty acids. Therefore, we hypothesized that longer chain docosahexaenoic acid (C22:6n-3) can more efficiently inhibit the protein degradation by regulating PPAR*γ/*NF*κ*B pathway in C2C12 myotubes than EPA.

In the present study, C2C12 myotubes were treated with EPA and DHA for 24 h, respectively. Meanwhile, knockdown of PPAR*γ* in C2C12 myotubes was achieved by RNA interference (RNAi). C2C12 myotubes for PPAR*γ* knockdown were also treated with EPA or DHA for 24 h, respectively. The actions of EPA or DHA were compared with those of a fatty acid-free control (containing BSA). The aim of this study was to investigate whether DHA can more efficiently inhibit protein degradation than EPA by regulating I*κ*B*α/*NF*κ*B pathway in C2C12 myotubes in a PPAR*γ*-dependent manner.

## 2. Materials and Methods

### 2.1. Materials

Cell culture media and supplements were obtained from Invitrogen (Carlsbad, CA, USA). Reagents for complementary DNA synthesis and the LightCycler system were obtained from Roche Applied Science (Mannheim, Germany). DHA, docosahexaenoic acid (C22:6n-3), EPA (C20:5n-3), essentially fatty acid-free BSA, monoclonal antiphospho-I*κ*B*α* (Ser32) antibody, and anti-I*κ*Ba antibody were obtained from Cell Signaling Technology, Inc. (Beverly, MA, USA). Antibodies against NF-*κ*B p65 were obtained from Santa Cruz Biotechnology (Santa Cruz, CA, USA). Finally, [*γ*-^32^P]ATP was obtained from Hartmann (Braunschweig, Germany).

### 2.2. Cell Culture

Mouse C2C12 myoblasts (American Type Culture Collection, Manassas, VA, USA) were maintained in Dulbecco's modified Eagle's medium supplemented with 10% fetal bovine serum, penicillin (50 units/mL), and streptomycin (50 mg/mL). When cells reached confluence, the medium was transferred to the differentiation medium containing Dulbecco's modified Eagle's medium and 2% horse serum, which was changed every other day. After four additional days, the differentiated C2C12 cells had fused into myotubes.

### 2.3. Transfection of Stealth RNAi for PPAR*γ* Knockdown in C2C12 Myotubes

Transfection of Stealth RNAi for PPAR*γ* knockdown in C2C12 myotubes was according to Kim et al. [14]. The PPAR*γ* Stealth Select RNAi oligonucleotide (Target Accession nos. NM 138712.1, M015869.2, NM138711.1, and NM 005037.3) was synthesized by Invitrogen. The Stealth RNAi negative control duplex (Invitrogen Corp., Carlsbad, CA, USA) was used as a control oligonucleotide. Transfection efficiency was monitored using a fluorescent oligonucleotide (BLOCK-iT Fluorescent Oligonucleotide; Invitrogen) and estimated to be 40% in C2C12 cells. The Stealth RNAi molecules were transfected into C2C12 myotubes using LipofectAMINE 2000 by following Invitrogen's protocols. The final concentration of 50 nM PPAR*γ* Stealth Select RNAi oligonucleotide was selected for C2C12 myotubes, and the Stealth RNAi oligonucleotides were transfected to the cells 48 h before the treatment of fatty acids. The ability of the Stealth RNAi oligonucleotide to knockdown PPAR*γ* expression was analyzed by western blot and real-time quantitative PCR on whole-cell extract.

### 2.4. Treatment  of  Cells

Lipid-containing media was prepared by conjugation of free fatty acids (DHA or EPA) with FFA-free bovine serum albumin, by a method modified from that described by Chavez et al. [15]. Briefly, FFAs were dissolved in ethanol and diluted 1 : 100 in DMEM containing 2% (w/v) fatty-acid-free bovine serum albumin. 3T3-L1 adipocytes and C2C12 myotubes were cocultured in serum free medium overnight and then washed with fresh medium, refed with medium containing the different treatments (BSA or EPA, resp.), and incubated at 37°C in 5% CO_2_ for 24 h before analysis. Cell viability was measured via trypan blue exclusion. At the end of the incubation, culture supernatant was collected and stored at −20°C until assayed.

### 2.5. Measurement of Protein Degradation in C2C12 Myotubes

Cells were analyzed for protein degradation using procedures adapted from Desler [16]. C2C12 myotubes were labeled on day 4 for 24 h with 1 *μ*mCi/mL of [^3^H]tyrosine before cells were washed, and the chase medium (DMEM + 2 mM tyrosine) was then added to wells and allowed to incubate for 4 h. Test medium was added and left for 20 h, and medium was sampled and counted. Remaining medium was aspirated off, and 1 mL of 0.5 M NaOH + 0.1% Triton was added. Plates were put in a cold room at 4°C overnight to allow the cells to dissolve. The cell suspension was placed in scintillation vials containing 5 mL of scintillation fluid, and wells were washed with 250 mL of 1 N acetic acid. This solution was added to scintillation vials and counted.

### 2.6. RNA Isolation

Total RNA was extracted using the TRIzol reagent (Invitrogen Corp., Carlsbad, CA, USA) according to the manufacturer's specifications. The RNA samples were quantified spectrophotometrically at 260 and 280 nm. The ratio of light absorbance at 260 nm to that at 280 nm was between 1.8 and 2.0, indicating that they were pure and clean. The quality of RNA was also checked by 1.0% agarose gel electrophoresis and staining with 1 *μ*g/mL ethidium bromide.

### 2.7. Reverse Transcription PCR and Real-Time Quantitative PCR Analysis

Reverse transcription (20 *μ*L) of total RNA (1 *μ*g) was performed using avian myeloblastosis virus reverse transcriptase with a first-strand cDNA synthesis kit for reverse transcription PCR. Aliquots (2 *μ*L) of the reverse transcription reactions were then submitted in duplicate to online quantitative PCR with the LightCycler 480 Real-Time PCR System (Roche Applied Science, Mannheim, Germany) with SYBR green using the FastStart DNA Master SYBR Green I. Initial real-time amplifications were examined by agarose gel electrophoresis, followed by ethidium bromide staining to verify that the primer pairs amplified a single product of the predicted size. Subsequent aliquots of the PCR reaction were checked by melting curve analysis as provided by the LightCycler system. Primer sequences and optimal PCR annealing temperatures (ta) are listed in Table 1. The PCR was performed in a volume of 20 *μ*L: 2 *μ*L of FastStart DNA Master SYBR Green I, 3 mM MgCl_2_ and primers according to a primer concentration of 1 *μ*M. The instrument settings were denaturing at 95°C for 10 min, 45 × denaturing at 95°C for 30 s, annealing at 59°C for 30 s, and elongation at 72°C for 8 min for (PPAR*γ* and *β*-actin). Quantification was performed by online monitoring for identification of the exact time point at which the logarithmic linear phase was distinguishable from the background. Serially diluted samples obtained by PCR with the above-mentioned primers from human myotubes were used as external standards in each run. The cycle numbers of the logarithmic linear phase were plotted against the logarithm of the concentration of the template DNA, and the concentration of cDNA in the different samples was calculated with the LightCycler software (version 5.32).

### 2.8. Isolation of Nuclear Extracts

Nuclear extracts were isolated according to Andrews and Faller [17]. Cells were scraped into 1.5 mL of cold phosphate-buffered saline, pelleted for 10 seconds, and resuspended in 400 *μ*L of cold BufferA (10 mM HEPES-KOH pH 7.9 at 4°C, 1.5 mM MgCl_2_, 10 mM KCl, 0.5 mM DTT, 0.2 mM PMSF, 5 *μ*g/mL aprotinin, and 2 *μ*g/mL leupeptin) by flicking the tube. Cells were allowed to swell on ice for 10 minutes and then vortexed for 10 seconds. Then, samples were centrifuged for 10 seconds and the supernatant fraction was discarded. Pellets were resuspended in 50 *μ*L of cold BufferC (20 mM HEPES-KOH pH 7.9 at 4°C, 25% glycerol, 420 mM NaCl, 1.5 mM MgCl_2_, 0.2 mM EDTA, 0.5 mM DTT, 0.2 mM PMSF, 5 *μ*g/mL aprotinin, and 2 *μ*g/mL leupeptin) and incubated on ice for 20 minutes for high-salt extraction. Cellular debris was removed by centrifugation for 2 minutes at 4°C and the supernatant fraction (containing DNA binding proteins) was stored at −80°C. Nuclear extract concentration was determined by using the Bradford method.

### 2.9. Electrophoretic Mobility Shift Assay

The transcription factor consensus oligonucleotides for the NF*κ*B responsive element (5′-AGT TGA GGG GAC TTT CCC AGG C-3′) and the AP1-responsive element (5′-CGC TTG ATG AGT CAG CCG GAA-3′) were purchased from Santa Cruz Biotechnology, Inc. (Santa Cruz, CA, USA). The probes were labeled with [*γ*-^32^P]-ATP using T4 polynucleotide kinase (Boehringer, Mannheim, UK) and purified on Sephadex G-25 (Pharmacia, UK) spin chromatography columns. For the binding reaction, nuclear extract (1 *μ*g of protein) was incubated in a 20 *μ*L volume with EMSA binding buffer (20 mM HEPES, pH 7.5; 50 mM NaCl; 1 mM EDTA; 1 mM dithiothreitol; 0.05% NP-40; 10% glycerol), 0.25 ng [*γ*-^32^P]-labelled probe, 1 mg/mL bovine serum albumin (Boehringer, Mannheim, UK), and 100 ng/mL poly d(I-C) for 20 min at room temperature. The specificity of the binding reaction was determined by coincubating duplicate samples with either 100-fold molar excess of unlabeled oligonucleotide probe or an anti-NF*κ*B antibody (anti-p65; Santa Cruz Biotechnology). Protein-nucleic acid complexes were resolved using a nondenaturing polyacrylamide gel consisting of 6% acrylamide gel run in 5 mM Tris, pH 8.3, and 38 mM glycine for 2 h at 200 V. Gels were transferred to Whatman 3M paper (Whatman, Inc., Clifton, NJ, USA), dried under a vacuum at 80°C for 1 h, and exposed to photographic film at −70°C with an intensifying screen.

### 2.10. Immunoblotting

In order to obtain total proteins C2C12, myotubes were homogenized in cold lysis buffer (5 mM Tris-HCl (pH 7.4), 1 mM EDTA, 0.1 mM phenylmethylsulfonyl fluoride, 1 mM sodium orthovanadate, and 5.4 *μ*g/mL aprotinin). The homogenate was centrifuged at 10,000 ×g for 30 min at 4°C. For obtaining total membranes from C2C12 myotubes, cells were collected into 10 mL of ice cold HES buffer (250 mmol/L sucrose, 1 mmol/L EDTA, 1 mmol/L phenylmethylsulfonyl fluoride, 1 *μ*mol/L pepstatin, 1 *μ*mol/L aprotinin, 1 *μ*mol/L leupeptin, and 20 mmol/L HEPES, pH 7.4) and subsequently homogenized at 4°C. After centrifugation at 1,000 ×g for 3 minutes at 4°C to remove large cell debris and unbroken cells, the supernatant was then centrifuged at 245,000 ×g for 90 min at 4°C to yield a pellet of total cellular membranes. Protein concentration was measured by the Bradford method. Proteins (30 *μ*g) were separated by SDS-PAGE on 10% separation gels and transferred to Immobilon Polyvinylidene difluoride membranes (Millipore, Bedford, MA, USA). Western blot analysis was performed using antibodies against phospho I*κ*B*α* (Ser32), I*κ*B*α*, and PPAR*γ* (Santa Cruz Biotechnology, Inc.). Detection was achieved using the EZ-ECL chemiluminescence detection kit (Biological Industries, Beit Haemek Ltd., Israel). Equal loading of proteins was assessed by red phenol staining. Size of detected proteins was estimated using protein molecular-mass standards (Invitrogen, Barcelona, Spain).

### 2.11. Statistical Analysis

Data were presented as means ± S.E. Differences between group means were determined by a one-way ANOVA using the computer program GraphPad Instat (version 2.03; GraphPad Software Inc., San Diego, CA, USA). When significant variations were found, the Tukey-Kramer multiple comparisons test was performed. Differences were considered significant at *P* < 0.05.

## 3. Results

### 3.1. Effect of Increasing Concentrations of n-3PUFA on Total Protein Degradation in C2C12 Myotubes

**Figure 1:**
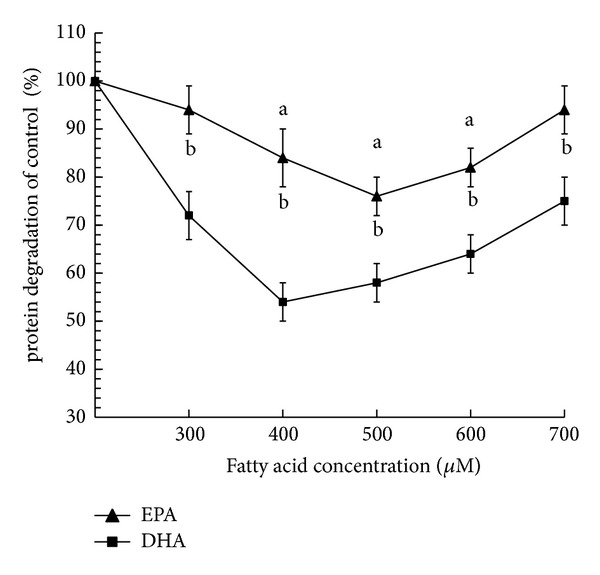
Effect of n-3 PUFA on total protein degradation in C2C12 myotubes. C2C12 myotubes were incubated (24 hours) with increasing concentrations of EPA (▲) or DHA (■), respectively. The radioactivity associated with cellular proteins is shown in the presence of 300 *μ*M, 400 *μ*M, 500 *μ*M or 600 *μ*M EPA, or DHA, respectively. Each treatment mean (*n* = 8) reported as a percentage of a FFA-free control; bars, SE. a, differences from controls in the presence of EPA (*P* < 0.01) and b, differences from the presence of DHA (*P* < 0.01). EPA = eicosapentaenoic acid, DHA = docosahexaenoic acid.

In the present study, two long chain n-3 PUFA were chosen for study: eicosapentaenoic acid (EPA), a C20:5n-3 n-3 polyunsaturated fatty acid, and docosahexaenoic acid (DHA), a C22:6n-3 n-3 polyunsaturated fatty acid. BSA was used as fatty acid-free control. C2C12 myotubes were incubated (24 hours) with 300 *μ*M, 400 *μ*M, 500 *μ*M, 600 *μ*M, or 700 *μ*M EPA and DHA, respectively. The effect of EPA or DHA on total protein degradation was dependent on the concentration added in the cell medium, as shown in Figure 1. Compared with the BSA control, EPA decreased total protein degradation when added at final concentrations of 400, 500, or 600 *μ*M for 24 hours (*P* < 0.01). The maximal effect was observed with 500 *μ*M of EPA. Compared with the BSA control, a 24 h incubation of C2C12 myotubes with 300 *μ*M, 400 *μ*M, 500 *μ*M, 600 *μ*M, or 700 *μ*M DHA, respectively, significantly decreased the total protein degradation (*P* < 0.01). Remarkably, the maximal effect was observed with 400 *μ*M of DHA.

### 3.2. Effect of 24 h Treatment with 400 *μ*M n-3PUFA on I*κ*B*α*/NF-*κ*B Pathway in C2C12 Myotubes

**Figure 2:**
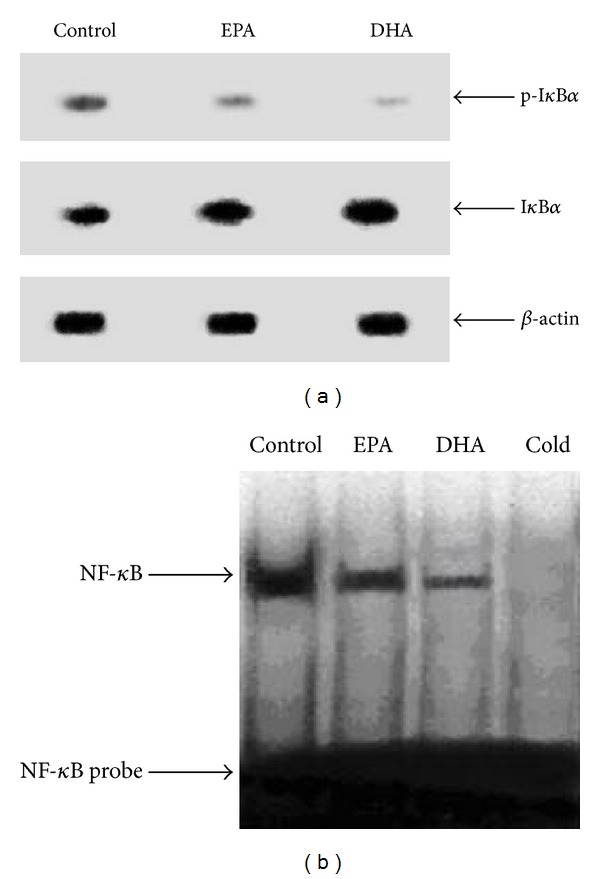
Effect of n-3 PUFA on the I*κ*B*α/*NF*κ*B complex in C2C12 myotubes. The C2C12 myotubes were incubated with 600 *μ*M BSA, 600 *μ*M EPA, or 600 *μ*M DHA for 24 hours, respectively. Protein extracts from C2C12 myotubes were assayed for western blot analysis with p-I*κ*B*α*, I*κ*B*α*, or *β*-actin (Figure 2(a)). The band on the western blot represented a protein with a molecular mass of ~37 kDa as determined by the molecular mass markers included in the experiment. Total nuclear protein was subsequently isolated and analyzed by EMSA for NF*κ*B DNA binding activity using a ^32^P-labeled double-stranded oligonucleotide of the NF*κ*B (Figure 2(b)). An additional unlabeled probe was added in the competition assay (cold). Data are representative of three experiments. BSA  =  bovine serum albumin, EPA  =  eicosapentaenoic acid, DHA  =  docosahexaenoic acid, and p-I*κ*B*α*  =  phosphorylated I*κ*B*α*.

After being treated with 400 *μ*M EPA, and DHA for 24 h in C2C12 myotubes, respectively, the levels of p-I*κ*B*α* and total I*κ*B*α* were measured by western blot method. The effect of 400 *μ*M EPA and DHA on the I*κ*B*α* protein level in C2C12 myotubes was present in Figure 2(a). As expected, compared with the BSA control, EPA (400 *μ*M, 24 h) decreased the I*κ*B*α* phosphorylation and caused approximately the 72% increase in the I*κ*B*α* protein level (*P* < 0.01). In addition, a 24 h incubation of C2C12 myotubes with 400 *μ*M DHA also decreased the I*κ*B*α* phosphorylation and caused approximately the 89% increase in the I*κ*B*α* protein level (*P* < 0.01). Taken together, these data suggested that 400 *μ*M DHA more effectively prevented the degradation of I*κ*B*α* by decreasing the phosphorylation of I*κ*B*α* and increased the I*κ*B*α* protein level in C2C12 myotubes than 400 *μ*M EPA.

To test whether incubation of C2C12 cells with 400 *μ*M EPA or DHA for 24 h affected NF*κ*B activity, we performed EMSA studies (Figure 2(b)). Compared with BSA, incubation of C2C12 myotubes with 400 *μ*M EPA for 24 h decreased the I*κ*B*α* phosphorylation and resulted in a 47% induction of the NF*κ*B DNA binding activity (*P* < 0.01). Furthermore, C2C12 myotubes incubated in the presence of 400 *μ*M DHA for 24 h decreased the I*κ*B*α* phosphorylation and caused a 68% reduction in the levels of the NF*κ*B DNA binding activity (*P* < 0.01). These results demonstrated that the inhibitory effect of 400 *μ*M DHA on the NF*κ*B DNA binding activity in C2C12 myotubes was greater than that of 400 *μ*M EPA.

### 3.3. Effect of 24 h Treatment with 400 *μ*M n-3PUFA on the Gene Expression of PPAR*γ* in C2C12 Myotubes

**Figure 3:**
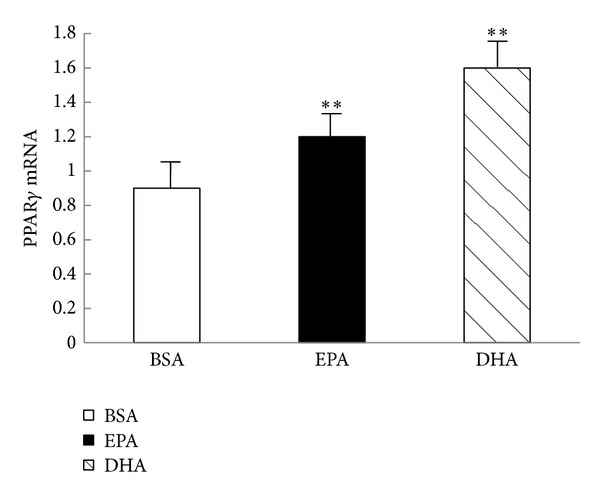
Effects of n-3 PUFA on *PPAR*γ** gene expression. C2C12 myotubes were incubated with 600 *μ*M EPA, 600 *μ*M DHA, or 600 *μ*M BSA for 24 hours. The *PPAR*γ** mRNA was determined using real-time PCR analysis and relative abundance of mRNA was calculated after normalization to *β*-actin. *PPAR*γ** = peroxisome proliferator-activated receptor *γ*, BSA  =  bovine serum albumin, DHA  =  docosahexaenoic acid, and EPA= eicosapentaenoic acid. Data are expressed as mean ± S.D. of 3 different experiments. **P < 0.01 versus control.

*PPAR*γ** gene expression data were presented in Figure 3. C2C12 myotubes incubated in the presence of 400 *μ*M EPA for 24 hours resulted in a 1.3-fold induction of the *PPAR*γ** expression (*P* < 0.01), whereas 24 h incubation period with 400 *μ*M DHA resulted in a 2.0-fold induction of the *PPAR*γ** expression (*P* < 0.01). In support of previous findings, 400 *μ*M DHA increased the *PPAR*γ** gene expression to a greater extent than 400 *μ*M EPA.

### 3.4. Effect of n-3PUFA on the I*κ*B*α*/NF-*κ*B Signaling Pathway and Total Protein Degradation in C2C12 Myotubes for PPAR*γ* Knockdown

To confirm that the inhibition of the I*κ*B*α*/NF-*κ*B signaling pathway and total protein degradation by n-3PUFA is mediated via activating the *PPAR*γ** mRNA expression, we examined the effect of n-3PUFA on the I*κ*B*α/*NF-*κ*B pathway in C2C12 myotubes for PPAR*γ* knockdown. 

**Figure 4:**
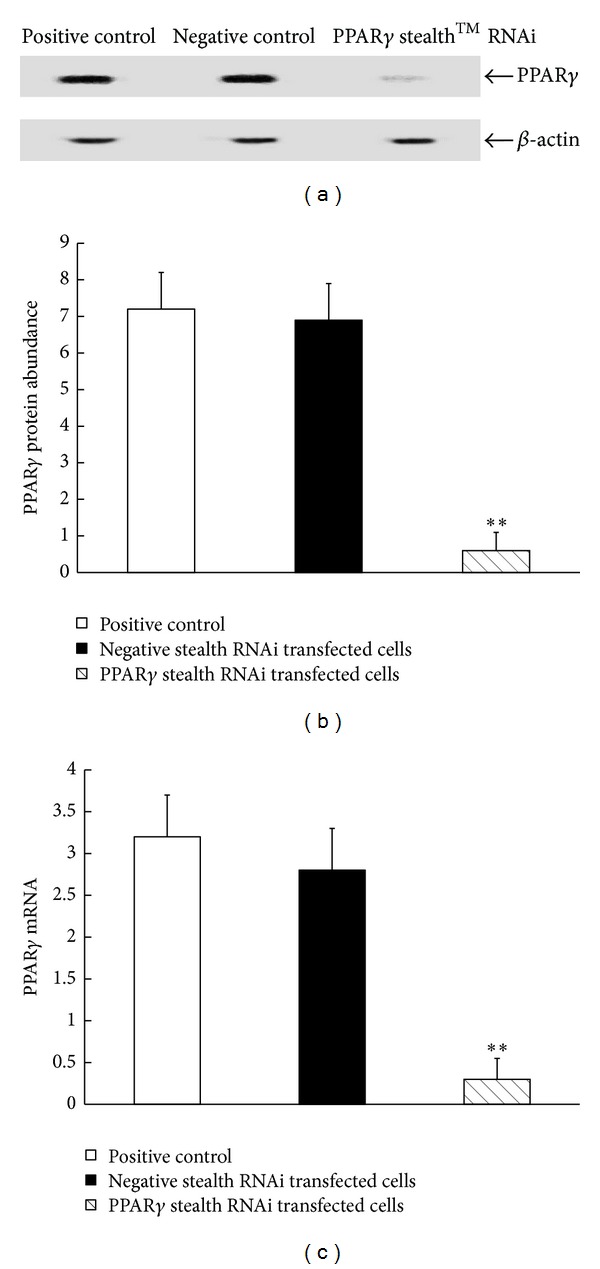
Transfection of Stealth RNAi for PPAR*γ* knockdown in C2C12 myotubes. The C2C12 myotubes transfected with either negative control Stealth RNAi oligonucleotide or PPAR*γ* Stealth RNAi oligonucleotide were incubated for 48 h, respectively. Protein extracts from C2C12 myotubes were assayed for western blot analysis with PPAR*γ* (Figure 4(a)). The band on the western blot represented a protein with a molecular mass of ~55 kDa as determined by the molecular mass markers included in the experiment. PPAR*γ* protein expression was determined by western blot and relative abundance of protein was calculated after normalization to *β*-actin (Figure 4(b)). The *PPAR*γ** mRNA was determined using real-time PCR analysis and relative abundance of mRNA was calculated after normalization to *β*-actin (Figure 4(c)). Data are expressed as mean ± S.D. of 3 different experiments. **P < 0.01 versus positive control group. PPAR*γ* = peroxisome proliferator-activated receptor *γ*.

The C2C12 myotubes transfected with either negative control Stealth RNAi oligonucleotide or PPAR*γ* Stealth RNAi oligonucleotide were incubated for 48 h, respectively. Transfection of Stealth RNAi for PPAR*γ* knockdown in C2C12 myotubes significantly decreased protein expression (Figures 4(a) and 4(b)) and PPAR*γ* mRNA (Figure 4(c)) to approximately 82% and 86% (*P* < 0.01), respectively. Negative control Stealth RNAi treatment had no influence on PPAR*γ* mRNA and protein expression (*P* > 0.05).

**Figure 5:**
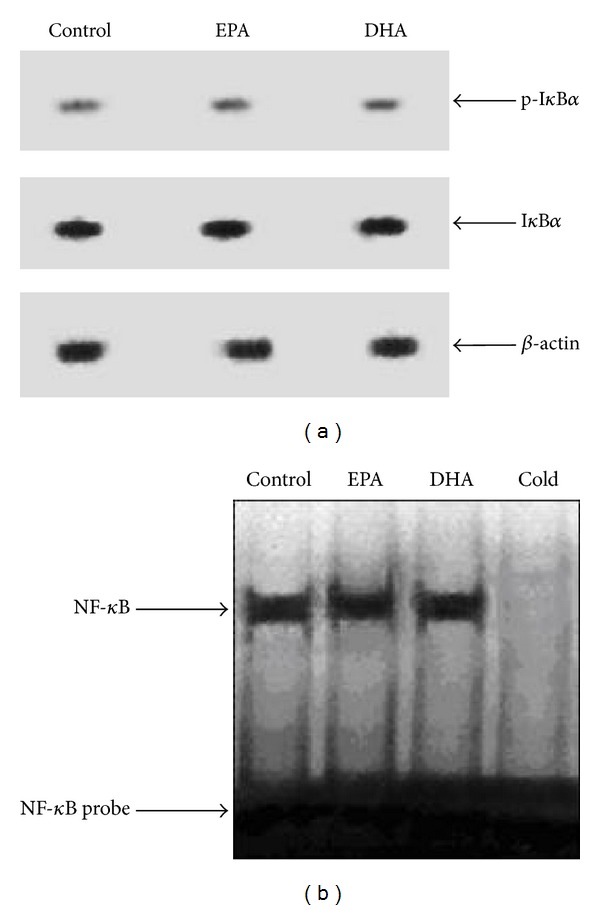
Effect of n-3 PUFA on the I*κ*B*α/*NF*κ*B complex in C2C12 myotubes knockdown of PPAR*γ*. The C2C12 myotubes transfected with PPAR*γ* Stealth RNAi oligonucleotide were incubated with 600 *μ*M EPA or 600 *μ*M DHA for 24 hours, respectively. BSA was used as fatty acid-free control. Protein extracts from C2C12 myotubes were assayed for western blot analysis with p-I*κ*B*α*, I*κ*B*α*, or *β*-actin (Figure 5(a)). The band on the western blot represented a protein with a molecular mass of ~37 kDa as determined by the molecular mass markers included in the experiment. Total nuclear protein was subsequently isolated and analyzed by EMSA for NF*κ*B DNA binding activity using a ^32^P-labeled double-stranded oligonucleotide of the NF*κ*B (Figure 5(b)). An additional unlabeled probe was added in the competition assay (cold). Data are representative of three experiments. BSA = bovine serum albumin, DHA = docosahexaenoic acid, EPA = eicosapentaenoic acid, and p-I*κ*B*α* = phosphorylated I*κ*B*α*.

**Figure 6:**
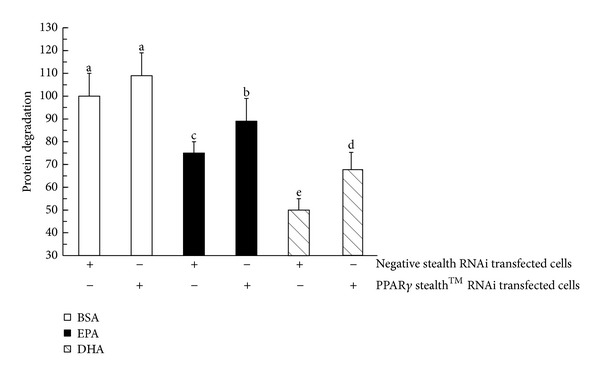
Effect of n-3 PUFA on protein degradation in C2C12 myotubes knockdown of PPAR*γ*. After the transfection of C2C12 myotubes with either the negative control Stealth RNAi oligonucleotide or the PPAR*γ* Stealth RNAi oligonucleotide for 48 h, C2C12 myotubes were incubated with 400 *μ*M EPA or 400 *μ*M DHA for 24 h. BSA was used as the fatty acid-free control. Data are expressed as mean ± S.D. of 8 different experiments. BSA = bovine serum albumin, DHA = docosahexaenoic acid, and EPA = eicosapentaenoic acid. Means without a common letter differ, *P* < 0.01.

In C2C12 myotubes transfected with PPAR*γ* Stealth RNAi oligonucleotide, treatment with 400 *μ*M EPA or 400 *μ*M DHA for 24 h did not affect the levels of p-I*κ*B*α* or total I*κ*B*α* (Figure 5(a)), NF*κ*B DNA binding activity (Figure 5(b)), compared with BSA (*P* > 0.05). Remarkably, Figure 6 showed that in C2C12 myotubes transfected with either negative control Stealth RNAi oligonucleotide, 24 h incubation period with 400 *μ*M EPA and DHA, respectively, caused 30% and 49% reduction in the rate of total protein degradation compared with BSA (*P* < 0.01). However, in C2C12 myotubes transfected with PPAR*γ* Stealth RNAi oligonucleotide, 24 h incubation period with 400 *μ*M EPA still caused a 17% reduction in the rate of total protein degradation (*P* < 0.01), whereas 24 h incubation period with 400 *μ*M DHA resulted in a 29% reduction in the rate of total protein degradation, compared with BSA (*P* < 0.01). 

## 4. Discussion

Previous studies have found that eicosapentaenoic acid (EPA, C20:5n-3) decreased gene expression of the muscle RING finger 1 (*MuRF1*), which has been demonstrated to have ubiquitin-ligase activity [13]. In the present study, it was further observed that total protein degradation was decreased by EPA at concentrations ranging from 400 *μ*M to 600 *μ*M and docosahexaenoic acid (C22:6n-3) at a wider dose range (300 *μ*M to 700 *μ*M), respectively. These results demonstrated that the effect of EPA and DHA on total protein degradation may be dependent on the concentration of fatty acids. Remarkably, in contrast to the fatty acid-free BSA control, the maximal effect for the inhibition of total protein degradation was observed at concentration of 500 *μ*M EPA and 400 *μ*M DHA, respectively. The results revealed that long chain DHA can more efficiently decrease total protein degradation than EPA. However, whether the suppressive effects of EPA and DHA on the total protein degradation depend on the chain length, it remains to be seen. 

Despite increasing evidence of the effect of long chain n-3 polyunsaturated fatty acid (n-3 PUFA) on total protein degradation, little is known concerning the mechanisms by which long chain n-3PUFAs regulate muscle protein degradation. As a result of 400 *μ*M EPA and DHA, both can inhibit total protein degradation. Therefore, in the current study, to test whether the effect of EPA or DHA on muscle protein degradation affected NF*κ*B activity, we investigated the effect of 400 *μ*M EPA or DHA on NF*κ*B activity. The key to NF*κ*B regulation is the I*κ*B*α* protein, which retain in the NF*κ*B cytoplasm. Phosphorylation of I*κ*B*α* by I*κ*B kinases triggers its polyubiquitinylation and degradation, thereby releasing NF*κ*B, which translocates to the nucleus. As expected, incubation of C2C12 myotubes with 400 *μ*M EPA for 24 h decreased the I*κ*B*α* phosphorylation and resulted in a 47% induction of the NF*κ*B DNA binding activity, whereas C2C12 myotubes incubated in the presence of 400 *μ*M DHA for 24 h decreased the I*κ*B*α* phosphorylation and caused a 68% reduction in the levels of the NF*κ*B DNA binding activity. These results demonstrated that DHA can more efficiently inhibit NF*κ*B activity than EPA. The results are in agreement with other studies showing that EPA inhibits NF*κ*B activation by preventing I*κ*B*α* phosphorylation and further reducing degradation of the inhibitory I*κ*B*α* protein [18, 19]. 

We then investigated the molecular mechanism by which EPA and DHA decreased NF*κ*B activation and inhibited total protein degradation. Many researches reported that activation of the transcription factor PPAR*γ* in the skeletal muscle can inhibit NF*κ*B activity [20, 21]. It was noteworthy that Li et al. [22] reported that n-3PUFA such as EPA and DHA are known natural ligands of PPAR**γ**, which were required in relatively high concentrations (approximately up to 100 *μ*mol/L) for PPAR activation. In the present study, C2C12 myotubes incubated in the presence of 400 *μ*M EPA for 24 hours resulted in a 1.3-fold induction of the *PPAR*γ** gene expression, whereas 24 h incubation period with 400 *μ*M DHA resulted in a 2.0-fold induction of the *PPAR*γ** gene expression. These results supposed that the effect of n-3PUFA on *PPAR*γ** gene expression is likely to depend on the chain length of fatty acids, which was in line with a previous report by Huang et al. [13], who found that C2C12 myotubes incubated in the presence of EPA (600 *μ*M, 24 h) compared with the BSA control resulted in a 1.47-fold induction of the* PPAR*γ** gene expression, while *α*-linolenic acid (ALA; C18:3n-3) with shorter chain lengths was not able to affect *PPAR*γ** gene expression. Taken together, these results supposed that long chain EPA and DHA can both decrease NF*κ*B activation and inhibit skeletal muscle degradation by activating *PPAR*γ** gene expression in a chain length dependent manner. However, because we did not test some other fatty acids, it is premature to give this conclusion without further study.

In order to investigate whether EPA inhibited the I*κ*B*α*/NF-*κ*B pathway and muscle protein degradation via activating the *PPAR*γ** mRNA expression, PPAR*γ* knockdown by RNA interference (RNAi) decreased PPAR*γ* mRNA and protein expression to approximately 86% and 82% in C2C12 myotubes. Interestingly, it was further observed that treatment with 400 *μ*M EPA or DHA for 24 h did not affect the levels of p-I*κ*B*α* and total I*κ*B*α* and NF*κ*B DNA binding activity in C2C12 myotubes for PPAR*γ* knockdown. These results demonstrated that PPAR*γ* knockdown by RNAi abolished the suppressive effects of the EPA or DHA on the I*κ*B*α/*NF*κ*B pathway in C2C12 myotubes, supporting that the EPA or DHA effects are mediated via PPAR*γ* activation. 

Remarkably, in contrast to the fatty acid-free BSA control, 24 h incubation period with 400 *μ*M EPA and DHA, respectively, can cause 30% and 49% reduction in the rate of total protein degradation in C2C12 myotubes without PPAR*γ* knockdown. However, in C2C12 myotubes for PPAR*γ* knockdown, 24 h incubation period with 400 *μ*M EPA still caused a 17% reduction in the rate of total protein degradation, whereas 24 h incubation period with 400 *μ*M DHA resulted in a 29% reduction in the rate of total protein degradation. The experiments founded that EPA and DHA both still decreased the total protein degradation in C2C12 myotubes, although PPAR*γ* knockdown by RNAi attenuated the suppressive effects of the EPA or DHA on the total protein degradation. These results revealed that the mechanism by which n-3PUFA regulated total protein degradation may be involved in other signaling pathway, except for the *PPAR*γ**/I*κ*B*α/*NF*κ*B signalling pathway in C2C12 myotubes.

Taken together, these results revealed that DHA, more efficiently than EPA, inhibited the protein degradation by regulating I*κ*B*α/*NF*κ*B signaling pathway in C2C12 myotubes by activating PPAR*γ* gene expression. 

## Figures and Tables

**Table 1:** Oligonucleotide polymerase chain reaction primers.

Gene^1^	Accession number	Source	Primer sequences (5′ → 3′)	Product size (bp)	ta^2^ (°C)	Amplification Efficiency (%)
*PPAR*γ**	NM011146	Mus	ATGGAGCCTAAGTTTGAGTT CAGCAGGTTGTCTTGGATGT	153	58	98
**β*-actin *	NM007393	Mus	CAGTGCGTGCTAAAGGGAGA CGCTCGTTGCCAATAGTGAT	148	59	97

^1^ *PPAR*γ*: Peroxisome proliferator-activated receptor *γ**.

^2^ta, optimal PCR annealing temperature.

## Abbreviations

ALA: *α*-Linolenic acid (C18:3n-3)
DHA: Docosahexaenoic acid (C22:6n-3)
EPA: Eicosapentaenoic acid (C20:5n-3)
EMSA: Electrophoretic mobility shift assay
BSA: Bovine serum albumin
MuRF1: Muscle RING finger 1
n-3PUFAs: N-3 polyunsaturated fatty acids
NF-*κ*b: Nuclear factor-kappa B
p-I*κ*B*α*: Phosphorylated I*κ*B*α*
PPAR*γ*: Peroxisome proliferator-activated receptor *γ*
RNAi: RNA interference.
